# IL28B Polymorphism Cannot Predict Response to Interferon Alpha Treatment in Patients with Melanoma

**DOI:** 10.1371/journal.pone.0112613

**Published:** 2014-11-12

**Authors:** Martin Probst, Christoph Hoeller, Peter Ferenci, Albert F. Staettermayer, Sandra Beinhardt, Hubert Pehamberger, Harald Kittler, Katharina Grabmeier-Pfistershammer

**Affiliations:** 1 Division of Immunology, Allergy and Infectious Diseases, Department of Dermatology, Medical University Vienna, Vienna, Austria; 2 Division of General Dermatology, Department of Dermatology, Medical University Vienna, Vienna, Austria; 3 Division of Gastroenterology and Hepatology, Department of Internal Medicine III, Medical University Vienna, Vienna, Austria; University of Tennessee, United States of America

## Abstract

**Background:**

Recent genome-wide association studies revealed the rs12979860 single nucleotide polymorphism (SNP) of the IL28B gene (CC genotype) to be the strongest pre-therapeutic predictor of therapy response to interferon alpha in patients with chronic hepatitis C infection. The favorable CC genotype is associated with significantly higher rates of sustained virologic response. No data exist on the role of IL28B polymorphism in interferon therapy of diseases other than viral hepatitis.

**Methods:**

A retrospective study involving 106 patients with melanoma who received low- or high-dose interferon therapy was performed. The CC and non-CC genotype of IL28B rs12979860 SNP were correlated with progression-free and overall survival.

**Results:**

44 (41.5%) patients were CC and 62 (58.5%) non-CC. There was no statistically significant difference in age at diagnosis, melanoma type or localization, Breslow level or AJCC stage between CC and non-CC patients. During the observation period (6.43±4.66 years) disease progression occurred in 36 (34%) patients after 5.5±4.3 years. 43.2% (19) of patients with CC and 27.4% (17) of patients with non-CC genotype were affected (p = 0.091). Disease progression was more frequent in patients on high dose interferon therapy and with a worse AJCC stage.

**Conclusion:**

In contrast to classical risk factors like tumor thickness and clinical stage, IL28B polymorphism was not associated with progression-free or overall survival in patients with melanoma treated with interferon alpha.

## Introduction

It is well established that melanoma represents a highly immunogenic tumor. Consequently various immune-modulatory approaches have been applied to cure melanoma or to delay disease progression. Immunotherapies aim at enhancing the immune response to malignant cells by increasing their immunogenicity or suppressing inhibitory pathways. Immunostimulatory mechanisms include interferon alpha treatment, interleukin-2 therapy, vaccination approaches and more recently blocking of inhibitory pathways with monoclonal antibodies directed against CTLA-4, PD-1 or PD-L1 [1].

Class I interferons enhance MHC and TAP expression, leading to improved antigen presentation in tumor cells [2], [3]. Furthermore, they modulate STAT1/STAT3 relation and likely have an impact on signal transduction in T cells [3], [4]. Interferon treatment has been associated with an increase in tumor infiltrating T cells and a reduction of regulatory T cells [5], [6]. Thus interferon is broadly used either in a low-dose protocol in stage IB/II melanoma or in a high-dose protocol for stage III melanoma. In several independent studies both regimens have been shown to improve progression –free survival but not overall survival [7], [8].

Interferon alpha treatment as adjuvant therapy of melanoma is a long-term therapy of 18 months and is often associated with severe side effects like liver, heart and bone marrow toxicity, fever and mood depression. This impairment of quality of life would make a predictive marker for the likeliness of therapy response very valuable and would also prevent unnecessary therapy costs.

Recently, a predictive marker has been identified for the probability of sustained virologic response after interferon alpha/ribavirin therapy in patients with chronic hepatitis C infection by genome wide association studies. A single nucleotide polymorphism (C/T dimorphism rs12979860) in the genomic region of IL28B, a type III interferon, is the strongest pre-therapeutic marker for success of therapy measured as sustained virologic response (SVR), i.e. negativity for HCV RNA 6 months post end of treatment. Whereas about 70% of patients with CC genotype reach this end point, SVR rates are about 32% and 23% in patients with CT or TT-genotype (i.e. non-CC genotype), respectively [9]–[11]. IL28B is involved in the induction of Interferon stimulated-genes (ISGs) and higher ISG activity in T allele carriers is thought to explain interferon resistance in hepatitis C [12], [13]. The impact of IL28B genotype has not been investigated in patients on interferon alpha treatment for indication other than viral hepatitis.

In this study we have analyzed a possible correlation between IL28B polymorphism and overall and disease free survival of stage IB/II and III melanoma patients who have received interferon alpha adjuvant therapy.

## Patients and Methods

### Patients

106 caucasian patients were included in this monocentric retrospective study conducted at the dermato-oncologic outpatient clinics of the Department of Dermatology, Medical University of Vienna. The eligibility criteria for enrollment in this study were (i) histologically proven melanoma stage IB, II or III and (ii) adjuvant low-dose or high dose interferon alpha therapy.

Patients' history, clinical and histological data were collected from patients' charts. Parameters analyzed included age, sex, type and location of melanoma, clinical stage at diagnoses, and disease progression under interferon therapy, disease-free survival and overall survival.

### Ethics

The study was approved by the Ethic committee of the Medical University Vienna (EK 1056/2011). Written informed consent was obtained from all patients who were alive at the time of enrollment in this study, but not of retrospectively analyzed deceased patients nor their relatives. Patients' data was anonymized and de-identified prior to analysis.

### IL28B Genotyping

Genomic DNA was isolated either from EDTA whole-blood samples with the QIAamp DNA Blood Mini Kit or formalin-fixed, paraffin-embedded tissue samples using QIAamp DNA FFPE Tissue Kit (Qiagen, Hilden, Germany). The rs12979860 SNP was analysed using the StepOnePlus Real Time PCR System (Applied Biosystems, Foster City, USA) in conjunction with a Custom TaqMan SNP Genotyping Assay developed together with Applied Biosystems. Sequences were obtained from the NCBI Entrez SNPDatabase (http://www.ncbi.nlm.nih.gov/sites/entrez). For rs12979860, oligonucleotides with the sequences 5′-GCCTGTCGTGTACTGAACCA-3′ and 5′-GCGCGGAGTGCAATTCAA-3′ were used as forward and reverse primer, respectively.

### Statistical analysis

Data were analyzed by IBM SPSS Statistics 22. We used the Mann-Whitney U test or Fisher's exact test for the comparison of groups as appropriate. Disease-free survival and overall survival were analyzed by the method of Kaplan-Meier, a Cox-regression model was used for multivariate analysis. For all statistical tests, a p-value <0.05 was considered statistically significant.

## Results

### Patient characteristics

62 (58.5%) male and 44 (41.5%) female patients were included in this study. The baseline characteristics are shown in table 1. Mean age at diagnosis was 50±14 years. The majority of patients were diagnosed with a nodular (41.5%) or superficial spreading melanoma (27.9%). In male patients melanomas were predominately located on the trunk (56.9%), while in female patients melanomas were equally distributed on the trunk and on the lower extremities (31.7% and 26.8% respectively). Mean Breslow index was 3.2±2.3 mm with a trend to higher levels in male and in older patients.

**Table 1:** Baseline characteristics of patients.

		n (%)
Sex	male	62 (58.5)
	female	44 (41.5)
Age		50.3±14 a
Histology	NMM	44 (41.5)
	SSM	29 (27.9)
	other	33 (30.6)
Breslow		3.2±2.3 mm
AJCC Stage	I	21 (19.8)
	II	49 (46.2)
	III	32 (30.2)
	unknown	4 (3.8)
IFN Therapy	Low dose	92 (86.8)
	High dose	14 (13.2)

95 of 106 (89.6%) patients underwent sentinel lymph node biopsy which was positive in 26 (27.4%) patients. 8 patients showed additional lymph node metastasis in subsequent lymph node dissection. Thus, according to AJCC classification, 19.8%of the patients were staged IB, 29.2% IIA, 14.2% IIB, 2.8% IIC and 30.2% III (table 1). Patients with lymph node metastasis had a trend to thicker tumors (4.2±3.3 mm versus 2.9±1.8 mm, p = 0.08) and a higher risk of disease progression (38.5% versus 27.5%, p = 0.05).

92 (86.8%) patients received low-dose interferon alpha, 14 (13.2%) high-dose interferon alpha therapy (table 1). Low-dose interferon was applied for a mean of 25.4±15.4 months, high dose interferon for 12.6±3.9 months.

During the observation period (6.4±4.7 years) disease progression was seen in 36 of 106 (34%) patients. Disease progression occurred after 5.5±4.3 years and the mean overall survival was 7±4.7 years. Disease progression was seen more often in patients receiving high-dose interferon (8/14, 57.1% versus 28/92, 30.4%, p = 0.05) and in patients with higher AJCC stages (2/21, 6.2% stage I versus 13/32, 40.6% stage III, p = 0.05).

### Distribution of IL28B polymorphism in the study population

44 (41.5%) patients had a CC and 62 (58.5%) a non-CC genotype. Distribution of C and T alleles in the study population was within the Hardy-Weinberg-equilibrium (table 2) and similar to the distribution previously described for the Austrian population (13). 25 (40.3%) male and 19 (43.2%) female patients had a CC genotype (p = 0.768). Mean age in the CC group was 48.8±14.4 years and 51.3±14.4 years in the non-CC group (p = 0.38). 17 (38.6%) patients with nodular melanoma and 8 (27.6%) patients with superficial spreading melanoma had a CC genotype (p = 0.10). In CC and in non-CC patients tumors were mainly located to the trunk. There was no statistical significant association of IL28B polymorphism with sex, age at diagnosis, melanoma type, anatomic site or ulceration of the primary tumour. There was a trend towards a higher Breslow index in patients with CC genotype, however also this difference did not reach statistical significance (p = 0.15). Distribution of AJCC stage did not differ between CC and non-CC patients (I-20.9%, II- 41.9%, III-37.2% versus I-20.3%, II-52.5%, III-27.1%, p = 0.50, table 3).

**Table 2:** Distribution of IL28B genotype in study cohort and correlation with expected distribution according to Hardy-Weinberg equilibrium.

polymorphism	genotype	observed	expected	p-value
	CC	44	46	
**IL28B (SNP rs12979860)**	CT	52	47	0.474
	TT	10	12	

**Table 3:** Risk factors like sex, age, type of melanoma, tumour thickness and AJCC stage did not differ between CC and non-CC genotype.

		CC	nonCC	p-value
Sex	Male	40.3%	59.7%	0.768
	Female	43.2%	56.8%)	
Age		48.8±14.4 a	51.3±14.4 a	0.379
Histology	NMM	38.6%	61.4%	0.099
	SMM	27.6%	72.4%	
Breslow		3.86±2.99 mm	2.75±1.45 mm	0.147
AJCC	I	20.9%	20.3%	0.496
	II	41.9%	52.5%	
	III	37.2%	27.1%	

### Correlation of IL28B polymorphism and disease progression

43.2% of patients with CC genotype and 27.4% of patients with non-CC genotype experienced disease progression (p = 0.09). Mean progression free survival was 5.3±5.1 years in the CC and 4.8±3.6 years in the non-CC group (p = 0.92). Overall survival was 7.6±5.6 years for patients with CC and 6.5±3.9 in patients with non-CC genotype (p = 0.45). During the observation period 4 patients in the CC and 4 patients in the non-CC group died of melanoma (p = 0.61).

**Figure 1:**
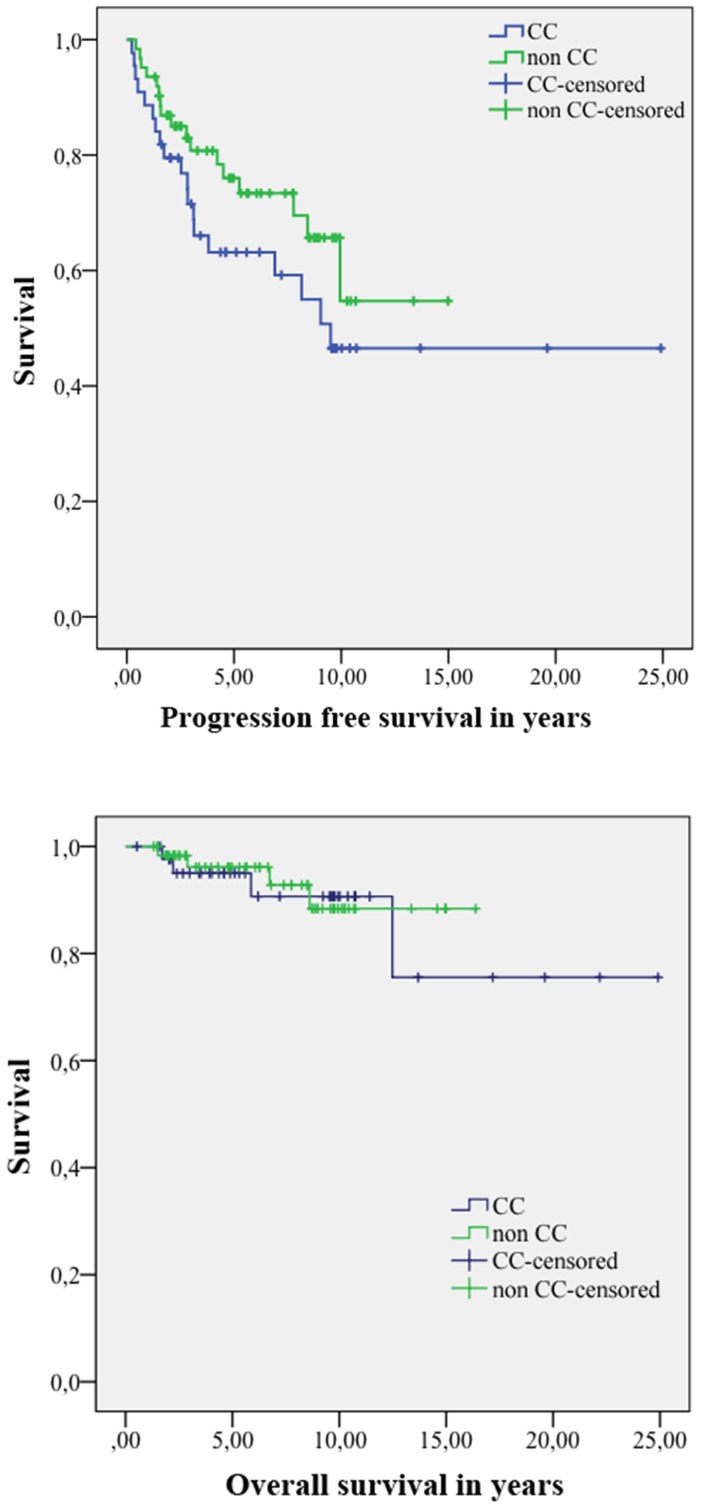
Correlation of IL28B polymorphism with progression-free and overall survival. A) Kaplan-Meier curve of progression-free survival according to IL28B polymorphism. No significant difference in progression-free survival could be observed for patients with CC or non-CC genotype (p = 0.176). Survival time on the x-axis is depicted in years. B) Kaplan-Meier curve of overall survival according to IL28B polymorphism. No significant difference in overall survival could be observed for patients with CC or non-CC genotype (p = 0.727). Survival time on the x-axis is depicted in years.

**Figure 2:**
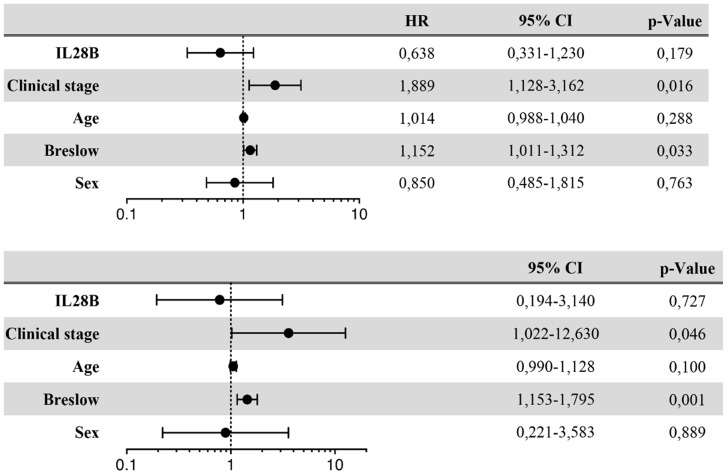
Univariate Cox regression analysis of risk factors associated with progression-free and overall survival. A) Risk factors associated with progression-free survival (univariate Cox regression analysis) B) Risk factors associated with overall survival (univariate Cox regression analysis).

**Figure 3:**
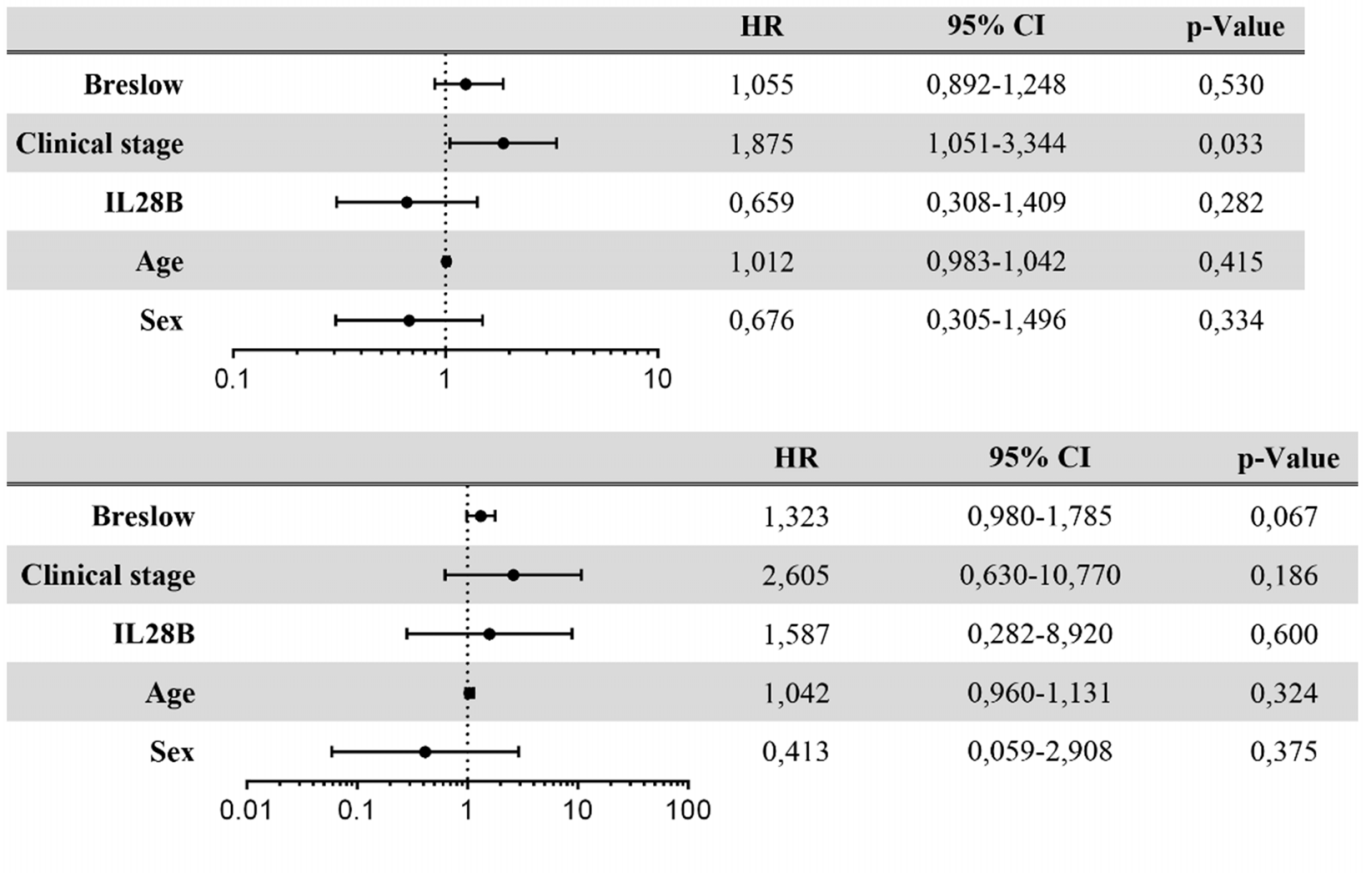
Multivariate Cox regression analysis of risk factors associated with progression-free and overall survival. A) Risk factors associated with progression-free survival (multivariate Cox regression analysis) B) Risk factors associated with overall survival (multivariate Cox regression analysis).

To assess an influence of IL28B polymorphism on survival of patients with melanoma treated with interferon alpha Kaplan-Meier analysis was done. There was no significant difference in progression free survival between CC and non-CC patients (Fig 1a). A similar result was seen when analyzing overall survival. Again, there was no impact of the IL28B polymorphism (Fig 1b). Applying univariate analysis, only tumor thickness and AJCC stage showed a statistically significant influence on progression and overall survival. There was no evidence for a correlation of IL28B polymorphism with progression free or overall survival (Fig 2). In multivariate Cox regression analysis only AJCC stage was significantly associated with disease-free survival (Fig 3a). Multivariate Cox analysis of overall survival did not reveal any statistically significant association when age, sex, IL28B polymorphism, Breslow index and AJCC stage were analysed together (Fig 3b). Since tumor thickness and AJCC stage are correlated multivariate Cox analysis was repeated separately with one of the two factors. In this analysis, a significant association of Breslow index and overall survival could be seen.

## Discussion

Personalizing or tailoring health care to the individual patient's need and genetic makeup gains more and more importance in daily practice. It is especially rewarding in the setting of therapies associated with severe side effects but also with high costs. Reliable biomarkers, that either predict response to therapy or risk of side effects, are a prerequisite for this approach. In case of unfavorable predictors addition or the sole use of adiuvants with no or only marginal side effects but also cost may be a valuable approach [14], [15].

Adjuvant interferon alpha treatment in melanoma fulfills the criteria for a therapeutic approach for which such a biomarker would be highly desirable: Several studies support the therapeutic efficacy in terms of disease-free survival and to a lower extent overall survival [16]–[21]. This potential benefit is counteracted by the fact that interferon alpha has to be taken for many months and is often associated with side effects that affect patients' quality of life.

Interferon alpha is used in several clinical settings. One main indication is the treatment of acute and chronic hepatitis C infection. Also in this application response to interferon is far below 100% and side effects are very common. However in this setting a biomarker – IL28 C/T dimorphism rs12979860 - predicting the probability to reach sustained virologic response (SVR) has recently been identified by genome-wide association studies [9]–[11]. Analysis of this SNP has become common practice and helps clinicians to either encourage their patients to undergo therapy or to defer treatment. Patients showing the favorable CC genotype have up to a twofold higher rate of SVR and higher rates of early virologic response than non-CC patients [22]. Another type III interferon, interferon λ4, was described recently as inducer of ISGs and has a similar prognostic value regarding treatment response [23].

The mechanism underlying the influence of IL28B polymorphism on the response to interferon alpha treatment is not known. IL28B is a type III interferon. Type I (interferon alpha) and type III interferon act via different receptors, but activate the same signal cascades and have similar effects [24]. IL28B polymorphism has been associated with IL28BmRNA expression levels and with activation levels of interferon responsive genes (ISG) [25], [26]. More recently, additional effects of the IL28B polymorphism like higher levels of KIR expression on NK cells in non-CC genotype and enhanced caspase activity in CC genotype have been described [27].

To our knowledge this is the first study which analysis the influence of the IL28B polymorphism on the success rate of interferon therapy in a disease other than viral infection.

In contrast to the abundant literature showing a clear cut effect of IL28B polymorphism on the therapy-outcome and SVR rates in HCV infected patients, we did not find evidence for a significant association in melanoma patients on adjuvant interferon alpha therapy.

The collective presented here is representative of melanoma patients. Several prognostic factors have been identified including tumor thickness, clinical stage but also other factors like e.g. melanin content have been shown to influence treatment response and have been correlated with overall and disease free survival [28]–[29]. Thus – as expected - well established risk factors like tumor thickness and clinical stage correlate with disease free and overall survival also in our study population.

There may be several explanations for the differing results in HCV and melanoma patients.

A recent study showed the correlation of ISG levels and the IL28B polymorphism is inversely correlated in healthy and HCV infected livers [30]. While in healthy livers CC genotype is associated with high mRNA levels of ISG, in the case of HCV infection, patients with the TT genotype show the highest levels of ISG. Thus in the setting of acute or chronic HCV infection the virus seems to induce a shift in the regulation of interferon signaling pathways resulting in a situation where patients with TT genotype already show maximal stimulated ISG levels, that might not be further enhanced by exogenous interferon, i.e. Interferon alpha therapy [31]. This is supported by other studies showing HCV related changes in IFN signaling pathways [32]–[34]. Therefore, virally induced changes in the interferon signaling cascade could be a prerequisite to reveal an influence of IL28B polymorphism.

Although it is generally accepted that anti-viral and anti-tumor immune responses share common mechanisms, there are also significant differences. Interferons are one of the most important antiviral defense mechanism. Thus, most viruses have developed anti-interferon escape mechanism. Modulation of interferon associated signaling pathways may therefore have much stronger implication for anti-viral than anti-tumor responses masking the effect of IL28B genotype.

Furthermore, while anti-viral immune response is directed to non-self, anti-tumor responses must deal with altered self and thus recognize subtle differences between healthy cells and tumors. In addition, tumor epitopes vary much more inter-individually than viral antigens. Also these individual differences could lead to a higher variability of the efficacy interferon treatment, independently of the IL28B polymorphism, making appreciation of its influence more difficult.

Our study has certainly several limitations.

On one hand we observed only a relatively low number of patients with progressive disease in this cohort. Furthermore, in contrast to HCV infection in melanoma IFN alpha therapy is an adjuvant treatment. While in hepatitis C sustained virologic response defined as absence of HCV-RNA 6 months post treatment can be used as end point, surrogate markers have to be used for melanoma. Thus, due to the natural course of the disease the observation time of this study is short and much longer observation periods might be needed.

In conclusion, we did not find any evidence of an association of IL28B polymorphism and treatment success with interferon alpha in patients with melanoma. IL28B polymorphism cannot be recommended as predictor in decision guidance in the same way as in hepatitis C.
